# Changing the HTS Paradigm: AI-Driven Iterative Screening for Hit Finding

**DOI:** 10.1177/2472555220949495

**Published:** 2020-08-18

**Authors:** Gabriel H. S. Dreiman, Magda Bictash, Paul V. Fish, Lewis Griffin, Fredrik Svensson

**Affiliations:** 1 The Alzheimer’s Research UK University College London Drug Discovery Institute, London, UK; 2 Department of Computer Science, University College London, London, UK

**Keywords:** machine learning, iterative screening, HTS, AI

## Abstract

Iterative screening is a process in which screening is done in batches, with each batch filled by using machine learning to select the most promising compounds from the library based on the previous results. We believe iterative screening is poised to enhance the screening process by improving hit finding while at the same time reducing the number of compounds screened. In addition, we see this process as a key enabler of next-generation high-throughput screening (HTS), which uses more complex assays that better describe the biology but demand more resource per screened compound. To demonstrate the utility of these methods, we retrospectively analyze HTS data from PubChem with a focus on machine learning–based screening strategies that can be readily implemented in practice. Our results show that over a variety of HTS experimental paradigms, an iterative screening setup that screens a total of 35% of the screening collection over as few as three iterations has a median return rate of approximately 70% of the active compounds. Increasing the portion of the library screened to 50% yields median returns of approximately 80% of actives. Using six iterations increases these return rates to 78% and 90%, respectively. The best results were achieved with machine learning models that can be run on a standard desktop. By demonstrating that the utility of iterative screening holds true even with a small number of iterations, and without requiring significant computational resources, we provide a roadmap for the practical implementation of these techniques in hit finding.

## Introduction

The current drug discovery paradigm is, to a large extent, focused on high-throughput screening (HTS), an approach in which large libraries of compounds are screened against the target of interest to identify suitable starting points for development.^1,2^ The hit rate in a typical HTS is relatively low, typically less than 1% in most assays,^3^ requiring large compound libraries to generate a sufficient number of hits for drug development programs to progress. The size of these libraries results in a high cost of screening as well as long lead times for campaigns. It is not uncommon for a screening campaign’s costs to run into the hundreds of thousand dollars.

With the advent of more disease relevant, but also more complex, phenotypic readouts in screening,^4^ the cost per screened compound has often increased. In our experience, a cost in excess of $1.50 per well is not uncommon. Clearly, there is a need for methods that increase the return rate for these screens. In addition, more chemical space than ever is now easily available for purchase, and there is a desire to query an ever-increasing amount of chemical matter. Combining these two developments requires new methods that allow more efficient use of time and resources.

An iterative approach can be used as an alternative to the brute-force approach of screening the full library.^5^ In iterative screening, the results from the fraction of the library so far screened are used as the input to a machine learning agent, which generates predictions that are used to select the next screening subset.^6^ Iterative screening has been shown previously to greatly enhance the efficiency of HTS.^7^ A plethora of different approaches for iterative screening have been reported, and a detailed review is available elsewhere.^5^

An iterative approach has previously been impractical because of the high labor costs associated with manually cherry-picking compounds from a screening deck, but recent advances in screening automation have made custom selection of compounds more broadly feasible, paving the way for artificial intelligence in the form of machine learning to drive the screening decisions. There is also an intrinsic tradeoff between the optimal number of compounds selected for the next iteration for the machine learning agent (ideally, it would operate with a iteration size of one, updating the model and improving its predictive power with the results from a single compound) and the practical feasibility of the screen. Although some efforts have been made previously to streamline this process, for example, through the picking of plates rather than compounds,^8^ there is a need for a thorough evaluation of these methods in a practically feasible setting.

Earlier studies have shown that iterative screening can greatly improve the efficiency of screening, with a high portion of all active compounds found while screening only a small part of the library. In this study, we build on these previous results and discuss how these methods can be practically applied. We investigate both the influence of different machine learning algorithms and the effects of limiting the number and size of iterations to what we believe is practically feasible in most modern lab settings.

## Materials and Methods

### HTS Data and Compound Representation

HTS data sets were downloaded from PubChem and used as provided after removal of duplicated compounds IDs.^9^ We selected the data sets to have no fewer than 50,000 tested compounds and to represent a diverse set of assay technologies and targets. Compounds were assigned an active or inactive label based on the PubChem annotations; any ambiguous compounds were labeled inactive. The data sets used in this study are listed in **Table 1**.

**Table 1.** PubChem HTS Data Sets Used in This Study.

PubChem AID	Number of Active Compounds	Total Number of Compounds	Usage	Target	Technology
596	1391	69,668	Development	MAPT	Fluorescence
628	2179	63,656	Development	CHRM1	Fluorescence
893	5649	73,912	Development	Hadh2	Fluorescence
894	6428	148,481	Development	HPGD	Fluorescence
938	1794	72,026	Development	TSHR	Fluorescence
995	707	70,898	Development	MAPK1	AlphaScreen
449739	4230	104,728	Development	CACNA1H	Calcium fluorescence
624255	4582	76,537	Development	*Trypanosoma cruzi* proliferation	Luminescence
1345083	6153	93,211	Development	Tox, HEK 293	Cell Titer Glo
598	5142	85,200	Validation	H69AR inhibition	Cell Titer Glo
488969	2166	105,151	Validation	Grm8	Calcium fluorescence
1259354	1804	75,924	Validation	IL1RL1	AlphaLISA

Compounds were represented using three different methods: extended connectivity fingerprints,^10^ chemical/physical descriptors, and molecular graphs. The combination of fingerprints and chemical/physical descriptors were used to train all methods except for the graph convolutional networks that used the molecular graphs. The fingerprints were 1024-bit Morgan fingerprints with radius 2 from RDKit.^11^ Ninety-seven chemical/physical descriptors were calculated with the RDKit as well, and these descriptors have previously been described and used with good results.^12^ Molecular graphs were constructed as PyTorch tensors.^13^ Each node (representing an atom) had 75 features.^14^

To evaluate the diversity of the hits, generic Murcko scaffolds were calculated using the RDKit (MurckoScaffold module). Generic scaffolds ignore atom type and bond type when identifying the scaffold.

### Machine Learning Methods

We applied a range of different machine learning algorithms: random forest (RF),^15^ support vector machine (SVM),^16^ light gradient boosting machine (LGBM),^17^ deep neural network,^18^ and graph convolutional neural network. All algorithms were implemented in Python using scikit-learn,^19^ lightgbm, PyTorch, and PyTorch Geometric.

For RF, SVM, and LGBM, a simple hyperparameter tuning was completed using scikit-optimize.^20^ Deep learning models were hand tuned with early stopping implemented on test/train loss curves. Detailed parameters used for the respective algorithm are shown in **Supplementary Table S1**. A central theme of HTS data is an extreme data imbalance, with active compounds composing a minority of all training examples.^21^ This was addressed by adjusting the loss contributions of each example.

### Iterative Screening Strategy

Each experiment starts with the initial iteration, consisting of 10% or 15% of the compound library selected using LazyBitVectorPick from RDKit’s MaxMinPicker module,^22^ which picks a diverse set of compounds from a random starting point. Based on the results of the screen on this initial compound set, a model is trained and used to predict the hit probability for remaining compounds in the library. This prediction is used in selecting the set of compounds for the next iteration. We evaluated iteration set sizes of 5% and 10% of the total library. Following each such iteration, the model is updated with the new information, and new predictions are made to select the next set of compounds.

After training on labeled data, models were used to generate probabilistic predictions for the activity of remaining unlabeled compounds. These predictions were ranked from high to low. To generate the list of compounds to be tested in the next iteration, a selection strategy operates on the ranked compounds. This strategy has two components. The first exploits the predictions to choose the compounds most likely to be hits for the next round of screening. The second explores the remaining compounds in the library to expand the model’s understanding of the behavior of untested compounds by randomly selecting compounds from this pool. For a given iteration with size X, the exploitation sample is of size 0.8X, and the exploration sample makes up the remaining component.

To estimate the robustness of the strategy, the entire iterative screening method was repeated three times for each data set, each time with different random starting points.

## Results

**Figure 1.**
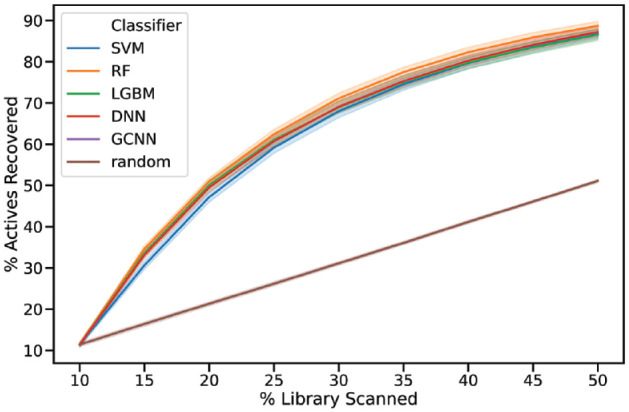
Mean recovery of active compounds versus percentage of library screened for different machine learning methods. An initial iteration of 10% were followed by steps of 5%. Shaded areas show the 68% confidence interval.

Based on 10% of the library as the initial batch, we evaluated the ability of different machine learning algorithms to recover actives across the nine different development data sets (**Table 1**). In each step, the algorithms selected an additional 5% of the library. The average retrieval is shown in **Figure 1**.

**Figure 2.**
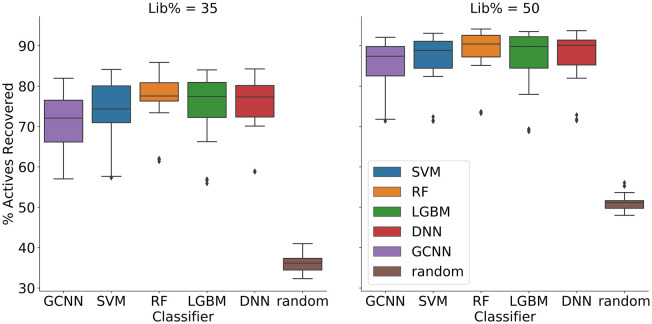
Percentage actives recovered with the respective machine learning algorithms at 35% (left) and 50% (right) of the library screened. An initial iteration of 10% was followed by steps of 5%. Plotted data include all three repeats for each data set.

The retrieval of active compounds at 35% and 50% of the library is shown in **Figure 2**. These results indicate that random forest had a slightly better performance on average across all data sets, retrieving a median of 78% of the active compounds (a full table of the average and median recovery is provided in the supplementary information).

Some variability between the different data sets was observed, with the best performing reaching 80% of actives recovered at 35% of the library screened and the worst only 55%. However, this lower recovery was observed for only one of the nine data sets (AID_628), which can be more clearly observed in **Supplementary Figure S1**.

**Figure 3.**
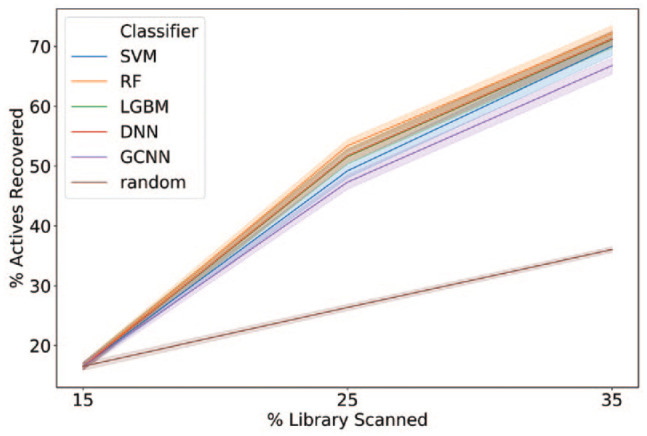
Mean recovery of active compounds versus percentage of library screened for different machine learning methods. An initial iteration of 15% was followed by steps of 10%. Shaded areas show the 68% confidence interval.

To investigate whether the number of iterations could be further reduced, we applied a strategy screening that used an initial batch of 15% of the library followed by two additional iterations of 10%. Again, RF was the best-performing algorithm, recovering a median of 71% of the active compounds at 35% of the library screened (**Figure 3**).

**Figure 4.**
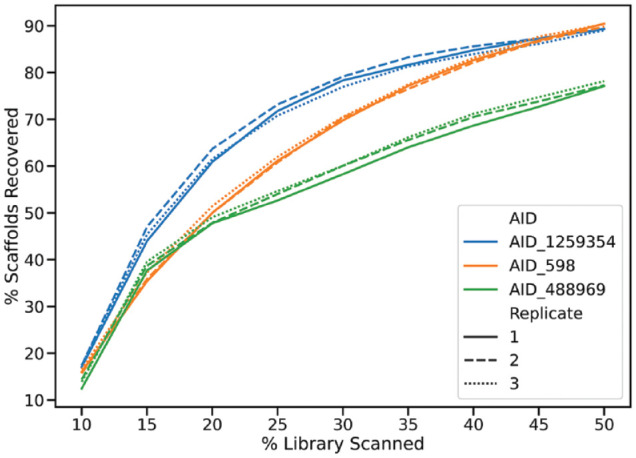
Recovery of Murcko scaffolds on the test data sets, three replicates were performed each with a set of starting compounds selected with LazyBitVectorPicker using a random starting seed. An initial iteration of 10% was followed by steps of 5%.

We also used three additional data sets (**Table 1**) to validate the best-performing setup (RF). The results confirmed the previous results with an average retrieval of 71% of the active compounds at 35% of the library screened when using a 10% of the library as the initial iteration followed by additional iterations of 5%. For these data sets, we also calculated the recovery of Murcko scaffolds^23^ to evaluate the hit diversity (**Figure 4**). The percentage of scaffolds recovered closely followed the recovery of active compounds.

## Discussion

Our results indicate that HTS can be greatly enhanced by the addition of iterative screening, in line with what has been shown previously.^5^ In our hands, the hit rate in the iterative screening was just greater than twice that of normal (random) screening, recovering a median of 78% of the active compounds when 35% of the library had been screened. We chose to focus on 35% of the library because this is a small enough fraction to make a large impact on the overall screening burden but at the same time allows for the identification of a large portion of the hits in our experiments. Evaluation of the hit diversity in terms of Murcko scaffolds also showed that we recovered diverse hits.

We wanted to design the approach to minimize the number of iterations required as this was deemed to pose the biggest practical limitation to the implementation of iterative screening. Based on our experience, up to three iterations is manageable without causing too much additional work in the form of compound picking and plating. This is fewer iterations than has been reported in most other iterative screening studies,^5,7,24^ although some examples exist.^25^ Using these settings, screening an initial 15% of the library followed by two additional iterations of 10%, we demonstrate that up to about 70% of the active compounds can be recovered while screening only 35% of the library. This represent a major saving of both cost and effort, especially for more advanced and costly assays, and it represents a level of improvement that, in our opinion, enables many more complex assay setups and provides the potential for better exploring chemical space.

Although smaller iterations give a higher retrieval of active compounds (78% vs. 71% when using 5% and 10% of the library in each iteration, respectively), we believe that three iterations of 15%, 10%, and 10% is a reasonable tradeoff in most settings. However, if maximal performance is sought, reducing the number of compounds screened in each iteration and increasing the number of iterations is recommended.

Similarly, if the objective is to reduce the number of screened compounds as much as possible, an even smaller library fraction should be considered. However, for these applications, other considerations become important, such as the diversity of the identified hits. Screening of a very small fraction might risk compromising the hit diversity despite enrichment of the total number of actives.

Clearly, these methods can be used for in-house compound collections, but perhaps more excitingly, they can be used to select compounds for each iteration to be purchased from a vendor catalogue. This not only circumvents the need for an in-house library and automated compound plating, making screening more accessible to academic (or other resource constrained) groups, but also unlocks access to a much larger chemical space for compound picking. The downside to using an external supplier is the lead time to source the new plates, resulting in a delay between iterations of up to a few weeks. An additional benefit to iterative screening methods, for both in-house and externally sourced libraries, is the potential to include various filters when selecting the compounds. If the library contains compounds that are undesirable for the project at hand, these can easily be excluded because the compounds are picked individually anyway.

Potential practical challenges remain and must be considered before embarking on an iterative screening campaign. Although good results can be obtained with just three iterations, there are logistical challenges with screening iteratively, as compound picking can be resource intensive and the interim analysis of screening data will potentially require more time for quality control and data management. If the lead time to produce the next iteration of plates is long, for example, if the compounds are ordered for each iteration, there is also a need for a process to maintain or reinstate cell cultures and to monitor assay performance. Although these are real issues, we believe that the increase of more cost-intensive assays will alter the balance in favor of iterative screening as compound-handling costs become dwarfed by other costs. In addition, the time requirement for some assays will be such that a full HTS cannot be enacted.

Throughout the iterative screening process, monitoring the process and evaluating whether the screening is on track are key. Because the difficulty in hit finding varies for different targets (variable hit rate), it is challenging to know a priori if sufficient hits will be generated for a machine learning approach to be efficient. For example, if after the first iteration of screening no hits have been identified, we would recommend either stopping the screening efforts or committing to screening the remainder of the library. An alternative approach is to try to leverage the continuous assay readout for machine learning, as there are examples of iterative paradigms using weak signals in the screening data to enrich actives in subsequent iterations.^26^ The performance of the iterative process can also be monitored and compared with the initial hit rate of the first batch; if the second iteration does not appear to deliver an increased hit rate, a switch to a full screen can be enacted.

Rewardingly, our experiments show that the method is not that sensitive to the selected machine learning algorithm (**Figures 1 and 2**). However, on average, RF had slightly better performance across the data sets. Recently, there has been substantial interest in deep learning methods for various predictive tasks, including applications in drug discovery.^27^ Although these methods might improve the predictions in certain settings, our results show that a deep learning method does not necessarily produce better results than more light-weight machine learning algorithms. This is, in many ways, good news, as methods such as RF are much faster to train and require less specialized knowledge to implement. Although we make no claims to have discovered the optimal method for iterative screening, the performance observed is more than sufficient to warrant the use of iterative screening. The method suggested in this article is able to retrain and predict the compounds for the next iteration in a matter of a few hours on most modern computers.

Iterative screening methods are sometimes not adopted because of concerns that hits will be missed when the whole library is not screened. Although this might be correct if all compounds that could ever be accessed were contained in the library, if other compounds could be considered, an iterative screening approach screening the same number of compounds as the initially considered library would almost certainly be far superior. Indeed, it is better to understand the benefits of iterative screening in terms of cost per hit. For any given budget, this method returns more than double the number of hits than can be expected using today’s HTS approach. This increased efficiency in terms of dollars per hit offers major benefits to small or resource-limited organizations. If a smaller number of compounds are screened with higher efficiency across an entire organization, that organization can pursue more programs with the same budget (in theory tripling the number of targets screened) while also significantly reducing the depletion of the compound library.

In conclusion, we show that iterative screening has matured to a point at which it is practically feasible to implement in the screening organization. Using well-established machine learning approaches, iterative screening can deliver significant boosts in screening efficacy and unlock more advanced and costly assays for large-scale screening.

## Supplemental Material

Dreiman_etal_SI – Supplemental material for Changing the HTS Paradigm: AI-Driven Iterative Screening for Hit Finding Click here for additional data file. Supplemental material, Dreiman_etal_SI for Changing the HTS Paradigm: AI-Driven Iterative Screening for Hit Finding by Gabriel H. S. Dreiman, Magda Bictash, Paul V. Fish, Lewis Griffin and Fredrik Svensson in SLAS Discovery
