# Supplementary material for: Changing the HTS Paradigm: AI-Driven Iterative Screening for Hit Finding
Source: SLAS Discov. 2020 Aug 18;26(2):257–62. doi: 10.1177/2472555220949495 (PMC7838329; doi:10.1177/2472555220949495)
Supplement: Dreiman_etal_SI – Supplemental material for Changing the HTS Paradigm: AI-Driven Iterative Screening for Hit Finding [file Dreiman_etal_SI.pdf]

Supporting Information

Changing the HTS Paradigm: AI Driven Iterative Screening for Hit Finding

Gabriel H. S. Dreiman[1,2], Magda Bictash[1], Paul V. Fish[1], Lewis Griffin[2], Fredrik Svensson[1*]

1. The Alzheimer's Research UK University College London Drug Discovery Institute, The Cruciform Building, Gower Street, London, WC1E 6BT, UK
2. Department of Computer Science, University College London, Gower Street, London, WC1E 6BT, UK

Table S1. Parameters and code snippets used with the different machine learning algorithms.

RF	RandomForestClassifier(n_estimators=1200, class_weight="**balanced**", max_features='**log2**',bootstrap=**True**,min_samples_split = 8, min_samples_leaf = 3, n_jobs = -1)
SVM	SGDClassifier(loss='**hinge**', penalty='**l2**', alpha=0.0001, l1_ratio=0.15, fit_intercept=**True**, max_iter=10000,tol=0.001, shuffle=**True**, verbose=0, epsilon=0.1, n_jobs=-1, random_state=**None**, learning_rate='**optimal**', eta0=0.0007, power_t=0.5, class_weight='**balanced**', warm_start=**False**, average=5)
LightGBM	lgb.LGBMClassifier(boosting_type='**dart**', num_leaves=42, max_depth=-1, learning_rate=0.25, n_estimators=1200, subsample_for_bin=200000, objective='**binary**', is_unbalance=**False**, max_bin=200, min_child_weight=0.001, min_child_samples=30, subsample=1.0, subsample_freq=0, colsample_bytree=1.0, reg_alpha=0.0, reg_lambda=0.0, random_state=**None**, n_jobs=-1, silent=**True**, importance_type='**split**')
GCNN	self.conv1 = GCNConv(n_features, 128, cached=**False**)<br>self.bn1 = BatchNorm1d(128)<br>self.conv2 = GCNConv(128, 64, cached=**False**)<br>self.bn2 = BatchNorm1d(64)<br>self.fc1 = Linear(64, 64)<br>self.bn3 = BatchNorm1d(64)<br>self.fc2 = Linear(64, 64)<br>self.fc3 = Linear(64, 1)<br>Loss: Categorical Crossentropy<br>Batch size: 128<br>Optimizer: adam<br>Epochs: 20
DNN	5 Dense layers,Units: [512,128,64,16,2], Activation: [Sigmoid,ReLu,ReLu,Relu,SoftMax], Loss: ‘Categorical Crossentropy’, Optimizer: adam, Batch-size: 500, Epochs: 5, Class weight: balanced with

	sklearn.utils.class_weight.compute_class_weight()

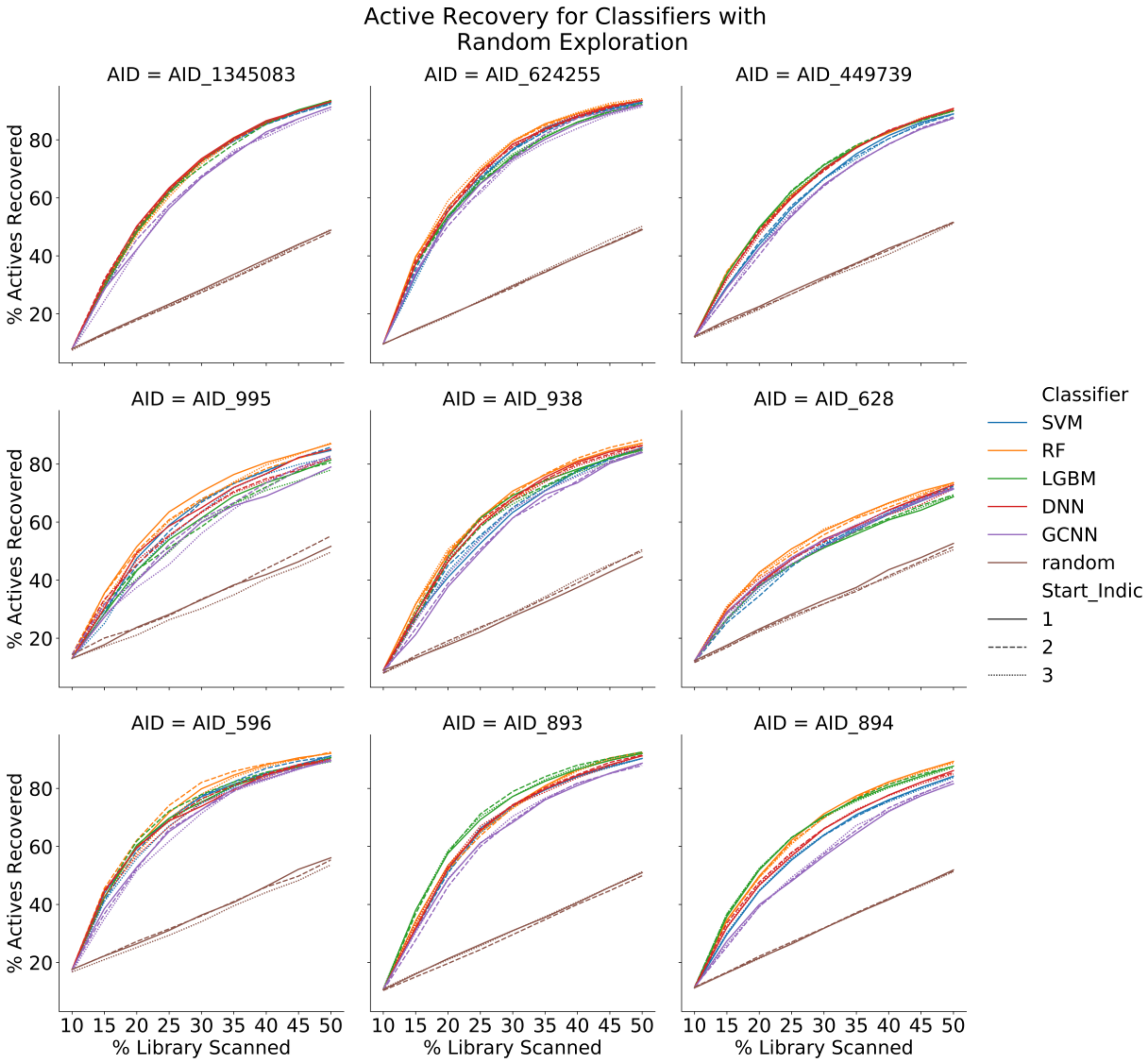

Fig S1. Active recovery curves for each AID with 10% start size and 5% iteration sizes. Three experiments were run for each AID, each experiment used a different random set of starting compounds. Within each experiment, each classifier was initially trained on the same set of starting compounds and subsequent selections were made based on its predicted scores for each iteration. Notice that AID_628 displays a different curvature than the other datasets, the low active recovery rates at both 35% and 50% are seen as outliers in the boxplots of Fig 2. Additionally, the performance of each model on different starting sets (represented by solid, large dashed, and small dashed lines) is relatively consistent for each AID. This consistency adds to our confidence that starting set selection has limited influence in the overall performance of the methods we present.

Table S2. Mean percentage of active compounds recovered across all experiments for each method for a 10% start and iterations of 5%.

Method	% of library screened	Mean % of actives recovered	Standard deviation
SVM	35 %	74.54	7.4
SVM	50 %	86.78	6.23
RF	35 %	77.49	6.63
RF	50 %	88.74	5.93
LGBM	35 %	75.11	8.09
LGBM	50 %	86.55	7.38
DNN	35 %	75.30	7.02
DNN	50 %	87.23	6.39
GCNN	35 %	71.40	6.98
GCNN	50 %	85.3	6.01
random	35 %	36.06	2.27
random	50 %	51.10	2.05

Table S3. Median percentage of active compounds recovered across all experiments for each method for a 10% start and iterations of 5%.

Method	% of library screened	Median % of actives recovered
SVM	35 %	74.35
SVM	50 %	88.87
RF	35 %	77.54
RF	50 %	90.43
LGBM	35 %	77.45
LGBM	50 %	89.83
DNN	35 %	77.30
DNN	50 %	90.07
GCNN	35 %	72.08
GCNN	50 %	87.42
random	35 %	36.15
random	50 %	51.16
